# Assessment of quaternary aquifer pollution sensitivity using modified DRASTIC models around stressed Canal in Eastern Nile delta, Egypt

**DOI:** 10.1038/s41598-025-09489-8

**Published:** 2025-07-13

**Authors:** Zenhom E. Salem, Abdullah M. Attiah, Abdelazim Negm, Mohamed S. Fathy, Youssef M. Youssef

**Affiliations:** 1 Geology Department, Faculty of Science, Tanta University, Tanta, Egypt; 2 Central Laboratory for Elemental and Isotopic Analysis (CLEIA), Nuclear Research Center (NRC), Egyptian Atomic Energy Authority (EAEA), Cairo, Egypt; 3 Water and Water Structures Engineering Department, Faculty of Engineering, Zagazig University, Zagazig, 44519 Egypt; 4 Geological and Geophysical Engineering Department, Faculty of Petroleum and Mining Engineering, Suez University, Suez, 43518 Egypt

**Keywords:** Nile delta, Quaternary aquifer, Groundwater vulnerability, Land uses, DRASTIC system, Sensitivity analyses, Environmental sciences, Natural hazards

## Abstract

Groundwater is generally less prone to contamination than surface water; however, pollutant infiltration can occur due to aquifer characteristics and anthropogenic land use (LU) changes. This study presents the first DRASTIC-based groundwater contamination risk (GwCR) framework for stressed aquifers around the Ismailia Canal, a newly developed artificial canal in Egypt. It evaluates the standard DRASTIC, Pesticide DRASTIC, DRASTIC-Lu, and Pesticide DRASTIC-Lu models, along with their modified versions, using Single Parameter Sensitivity Analysis (SPSA) and GIS techniques. SPSA identified the following parameter weights for the pesticide-specific DRASTIC model: D > S > T > A > I > C > R. One- Map Removal Sensitivity Analysis (MRSA) analysis showed the Pesticide DRASTIC model was most sensitive to net recharge (1.36%) and soil media (1.0%), with moderate sensitivity to the vadose zone (0.65%), topography (0.45%), and hydraulic conductivity (0.42%). Excluding key parameters, particularly D, A, and S, caused significant variability, impacting vulnerability assessments. The Pesticide DRASTIC_SPSA_ model outperformed others, with 82.6% of groundwater samples, along with relative frequency greater than 0.8 in moderate to very high vulnerability zones. The Pesticide DRASTIC_SPSA_ map indicated that 36.21 km², 6.26 km², 19.03 km², 31.0 km², and 13.09 km² of the study area were in very high, high, moderate, low, and very low susceptibility zones, respectively. The high and very high vulnerability zones were primarily located in the northern and southern regions of the Ismailia Canal, where the protective clay layer is absent and shallow groundwater and sandy vadose zones prevail. The very high vulnerability area increased from 27.3 km² in the original DRASTIC model to 30.52 km² under the Pesticide DRASTIC_SPSA_ model. These findings apply to other regions with similar hydrogeological and socio-economic conditions, offering insights for future freshwater canal system development in Egypt.

## Introduction

 Sustainable development challenges often stem from the availability and suitability of freshwater resources, particularly when surface water sources are limited or polluted^1,2^. In North African developing nations, particularly arid regions like Egypt, the scarcity of freshwater has heightened the focus on utilizing groundwater to meet diverse needs^3^. While groundwater is generally more accessible, reliable, and less prone to contamination compared to surface water, it is still susceptible to pollutants that can degrade its quality^4–9^. Additionally, population growth has amplified the need to identify and secure vital groundwater reserves; however, these resources are increasingly threatened by salinization driven by rising urban water demand^10^. For example, contaminants originating from unregulated agricultural and aquaculture practices have led to the degradation of both surface and groundwater quality^11^. Consequently, groundwater contamination remains a critical issue in rapidly developing regions, where economic growth often clashes with environmental protection, jeopardizing water quality and availability^3,10,12^.

Studies have extensively examined chemical pollutants in groundwater^13,14^, revealing significant impacts from human activities, including agricultural runoff, industrial effluents, improper waste disposal, and inadequate wastewater treatment^15^. Natural hydrological and geological conditions can further exacerbate aquifer contamination, increasing groundwater vulnerability^16^. Shallow aquifers are especially prone to pollution, highlighting the need for effective protection strategies^17,18^. These stressors threaten ecosystem integrity, necessitating a balance between current water demands and resource sustainability^19^. Therefore, maintaining a sustainable groundwater supply and efficient irrigation practices is essential for food security and long-term economic and social development.

Groundwater vulnerability assessments are essential for managing aquifer risks, as they indicate contamination susceptibility based on hydrogeological characteristics and overlying strata^20,21^. These assessments evaluate the likelihood of contaminants reaching the water table by considering geological structure, aquifer hydraulics, and soil properties^10,22,23^. Vulnerability maps visually classify areas to aid in groundwater protection and sustainable management. Such assessments support planners and decision-makers in socio-economic planning for urban development, land reclamation, and industrial projects^24–26^. Using the DRASTIC system, the groundwater vulnerability of an aquifer can be systematically assessed based on its hydrogeologic settings that represent the major geologic and hydrologic characteristics of the aquifer^24^.

Various index-based methods have been globally employed to assess groundwater contamination risk (GwCR), including DRASTIC^24^, GOD^27^, SINTACS^28^, and GALDIT^29^. Among these, DRASTIC is one of the most widely applied and adaptable methods for evaluating groundwater vulnerability across diverse hydrogeological contexts^30–33^. By integrating geological, geomorphological, and hydrogeological factors, DRASTIC offers a comprehensive analysis^34^. Specifically, it assesses groundwater vulnerability using seven variables: Depth to groundwater (D), Net Recharge (R), Aquifer media (A), Soil media (S), Topography (T), Impact of the vadose zone (I), and Hydraulic Conductivity of the aquifer (C), particularly in urbanized riverine areas^17,35^. DRASTIC parameters are typically scored and weighted using multi-criteria decision analysis (MCDA) based on expert judgment within Geographic Information Systems (GIS) to evaluate groundwater vulnerability^31^. GIS is effective for implementing DRASTIC indexes, as it facilitates the integration of data layers, adjusts classification parameters, and manages spatial data for analysis and presentation^36–39^. In regions characterized by intensive agricultural activity, such as riverine landscapes, modified DRASTIC variants—such as pesticide DRASTIC—assign greater weights to parameters like soil type and slope (Banton and Villeneuve, 1989). The flexibility of the DRASTIC model allows for the calibration of weights and ratings to reflect local geological and hydrological conditions, thereby enhancing its accuracy and applicability to specific study areas^33^.

**Fig. 1:**
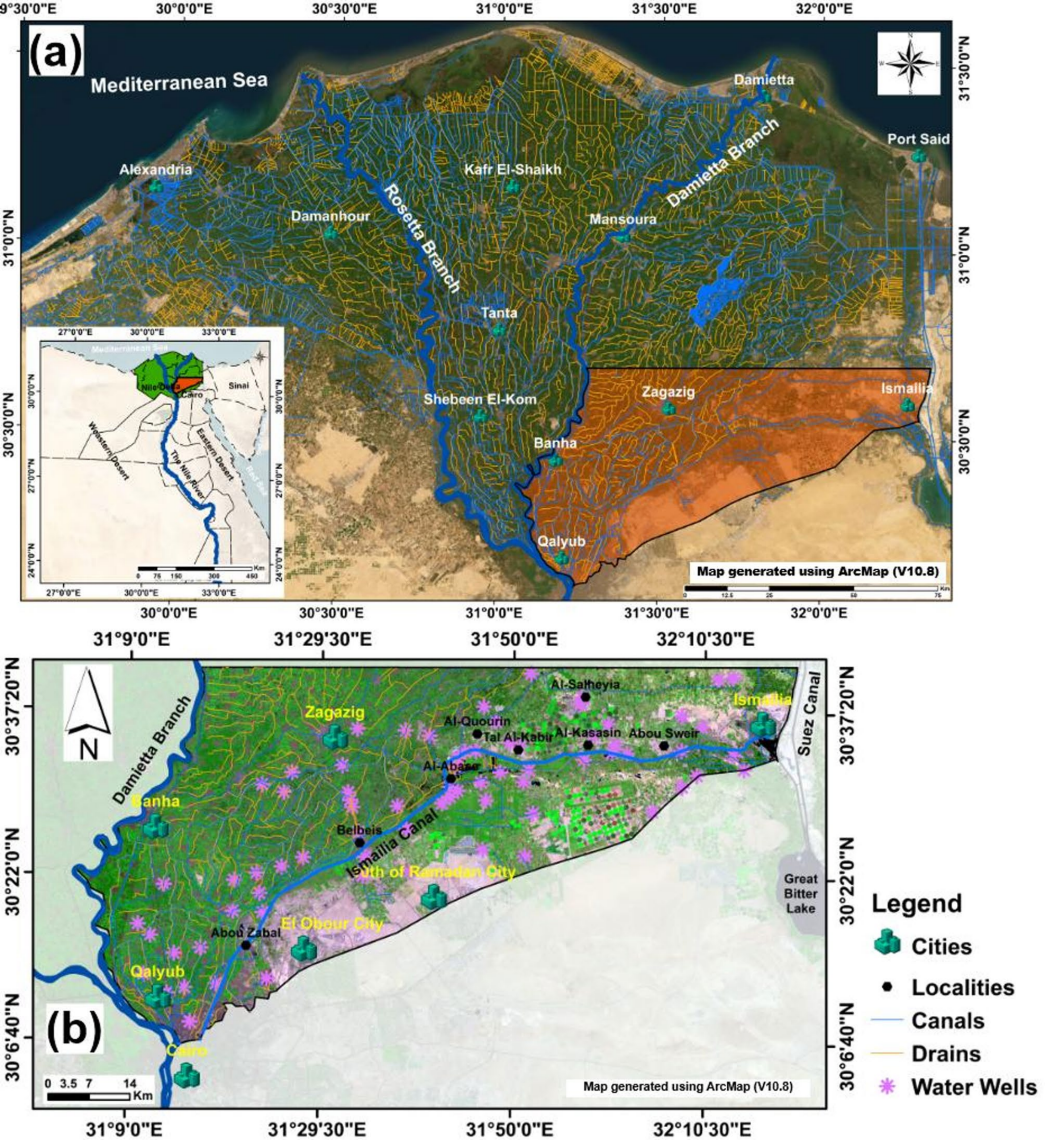
Study area location map: (**a**) Base map showing the administrative boundaries of Egyptian governorates with the Nile Delta highlighted in green, and (**b**) Landsat-8 image (RGB: 753) of the study area, overlaid with groundwater sampling locations.

The Eastern Nile Delta region in Egypt holds substantial socio-economic and environmental significance, functioning as a vital development corridor within the densely populated Nile Valley and Delta^40^. The Quaternary aquifer, depicted in Fig. 1A, serves as the principal source of groundwater in this area, supporting a wide range of human activities. The completion of the Ismailia Canal in 1862 marked a pivotal advancement, supplying essential irrigation and potable water resources to Egypt^41^. In recent decades, the newly urbanized zones adjacent to the Ismailia Canal in the eastern Delta have experienced intensive irrigation and agricultural expansion, characterized by the widespread application of fertilizers and pesticides^42^. Additionally, the continued reliance on traditional flood irrigation techniques has exacerbated the vulnerability of groundwater resources, as elevated infiltration rates heighten the potential for contamination^42–45^. While previous investigations have largely concentrated on groundwater chemistry, aquifer lithology, and the rising groundwater table, limited attention has been directed toward evaluating groundwater pollution or its susceptibility within this highly anthropogenically influenced setting. Comparable studies from other regions^34,36,46^, have emphasized the critical role of land use in shaping groundwater quality, demonstrating that varying land use practices and anthropogenic activities contribute to contaminant infiltration in riverine systems^46–49^. To address this gap, enhanced versions of the DRASTIC model—such as DRASTIC-Lu and Pesticide DRASTIC-Lu—have been used to incorporate land use (Lu) parameters, thereby improving the assessment of groundwater vulnerability in this stressed riverine canal. Accordingly, the present study futher employs the standard DRASTIC and Pesticide DRASTIC models to evaluate GwCR in the vicinity of the Ismailia Canal, generating vulnerability maps within a GIS-based framework. Furthermore, a comprehensive sensitivity analysis was conducted to examine the relationship between model input parameters and spatial variables, offering key insights into the model’s robustness and deepening the understanding of the primary factors influencing groundwater vulnerability. This study presents a robust qualitative and quantitative framework for assessing groundwater vulnerability, offering critical support for future planning and development of freshwater canal systems in Egypt under similar hydrogeological and socio-economic conditions. For example, it can inform the design and implementation of artificial agricultural canals within the New Delta megaprojects aimed at expanding agricultural productivity (https://sis.gov.eg/Story/162017/New-Delta-project?lang=en-us, accessed on March 25, 2025). The proposed approach also contributes to mitigating the adverse impacts of anthropogenic activities on groundwater resources.

## Study area

**Fig. 2:**
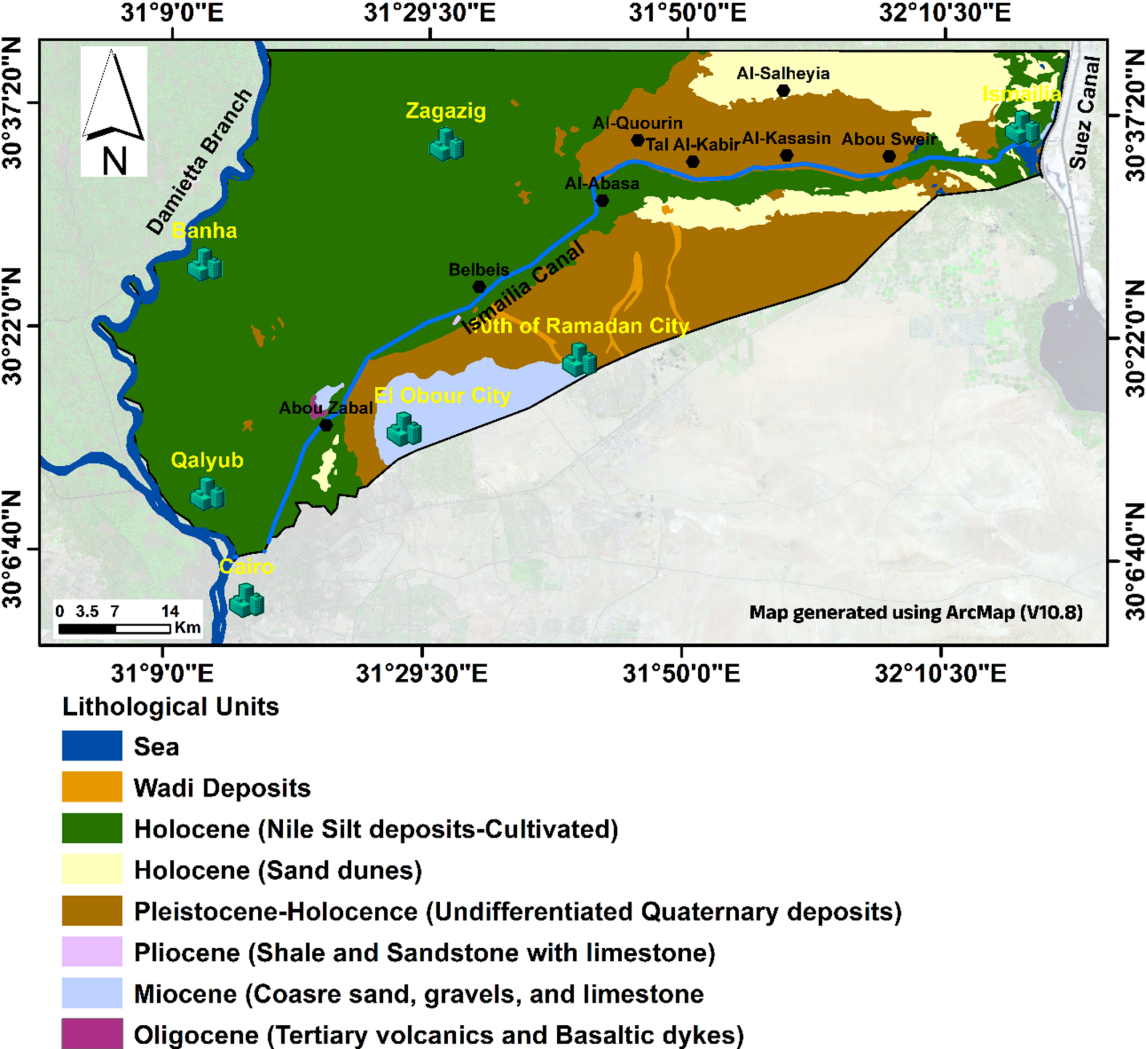
The geologic map of the study area (EGPC/CONOCO, 1987).

The study area is located in the Eastern Nile Delta, extending along and surrounding the Ismailia Canal, which flows from the River Nile in Cairo Governorate to El Temsah Lake in Ismailia Governorate^13,45^. It covers an area of approximately 4300 km² and lies between 30°03′ and 30°42′ N and 31°03′ and 32°24′ E (Fig. 1B). The area is bordered by the Suez Canal to the east and the River Nile and Damietta branch to the west, with the southern boundary defined by the desert rolling plains and foothills of the Cretaceous and Tertiary structural exposures (Fig. 2). Most of the region is covered by Quaternary deposits, which are classified into two rock units: the upper Bilqas Formation (Holocene Nile silt and clay) and the lower Mit Ghamr Formation^50–53^.

**Fig. 3:**
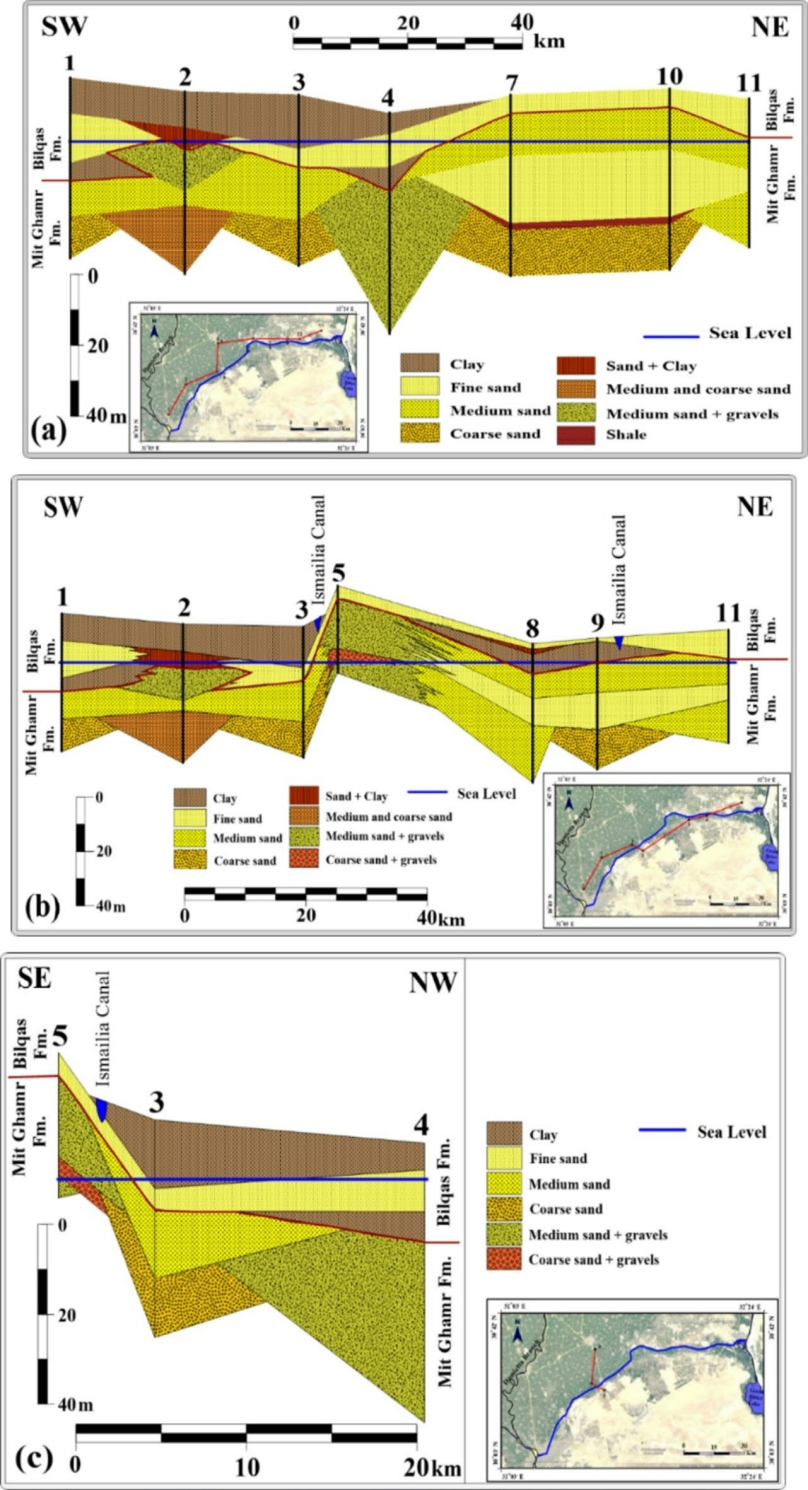
Geological cross-sections of the Quaternary Aquifer within the study area are presented as follows: (**A**) a SW–NE-oriented section approximately parallel to the Ismailia Canal; (**B**) a SW–NE-oriented section intersecting the Ismailia Canal at two locations; and (**C**) a SE–NW-oriented section. All cross-sections were constructed using Surfer software (Golden Software, Version 16.6.484), with sea level adopted as the datum plane.

Three stratigraphic cross-sections were constructed using columnar data from 11 wells, ranging in depth from 35 m to 70 m, located around and along the Ismailia Canal, to illustrate the geological setting of the Quaternary Aquifer (Fig. 3). The aquifer is characterized by two types of sediment: a sand-dominated layer in the eastern and southeastern regions, and a clay-dominated layer in the northern and northwestern regions (Fig. 3). As shown in Fig. 3A-B, wells 1, 2, 3, and 4, near the Damietta branch, have upper sections consisting of recent Nile fluvial deposits, while wells 5, 7, 10, and 11 lack clay and are primarily composed of sand deposits from desert and wadi sources. Wells 8 and 9, located away from the Nile but along an ancient river branch (Fig. 3b), show similar sand-dominated characteristics. In the western part of the study area, north of the Ismailia Canal, a clay-dominated sediment layer covers the surface, thinning and disappearing toward the east and northeast, as seen in wells 1, 2, 3, and 4, and vanishing completely in the eastern region (wells 7, 10, and 11) (Fig. 3A). The eastern part, both north and south of the Ismailia Canal, consists entirely of sand-dominated sediments, with potential clay interbeds, particularly near the canal where it crosses an ancient river branch, as shown in wells 5, 8, 9, and 11 (Fig. 3C). Conversely, the western part of the study area features a surface clay-dominated layer overlying sand-dominated sediments, which decreases in thickness toward the south of the Ismailia Canal, as seen in well 5 (Figs. 3B-C). Recharge in the region is derived from three primary sources: rainfall, infiltration from irrigation systems, and the movement of water through the Nile Valley aquifer. The Nile Delta experiences sparse and irregular rainfall during winter, amounting to less than 155 mm annually^54^. Since 1981, groundwater extraction has increased at an average linear rate of 0.1 billion cubic meters (Bm³) per year, with an accelerated rate of 0.2 Bm³/year observed between 2003 and 2010^44^. Youssef et al.^3^ reported a substantial decline in terrestrial water storage (ΔTWS) of −86.1 mm between 2005 and 2015, correlating with a reduction in surface water bodies. These trends are linked to extensive aquaculture developments and unauthorized extractions of both surface and groundwater^3,55^.

## Materials and methods

**Fig. 4:**
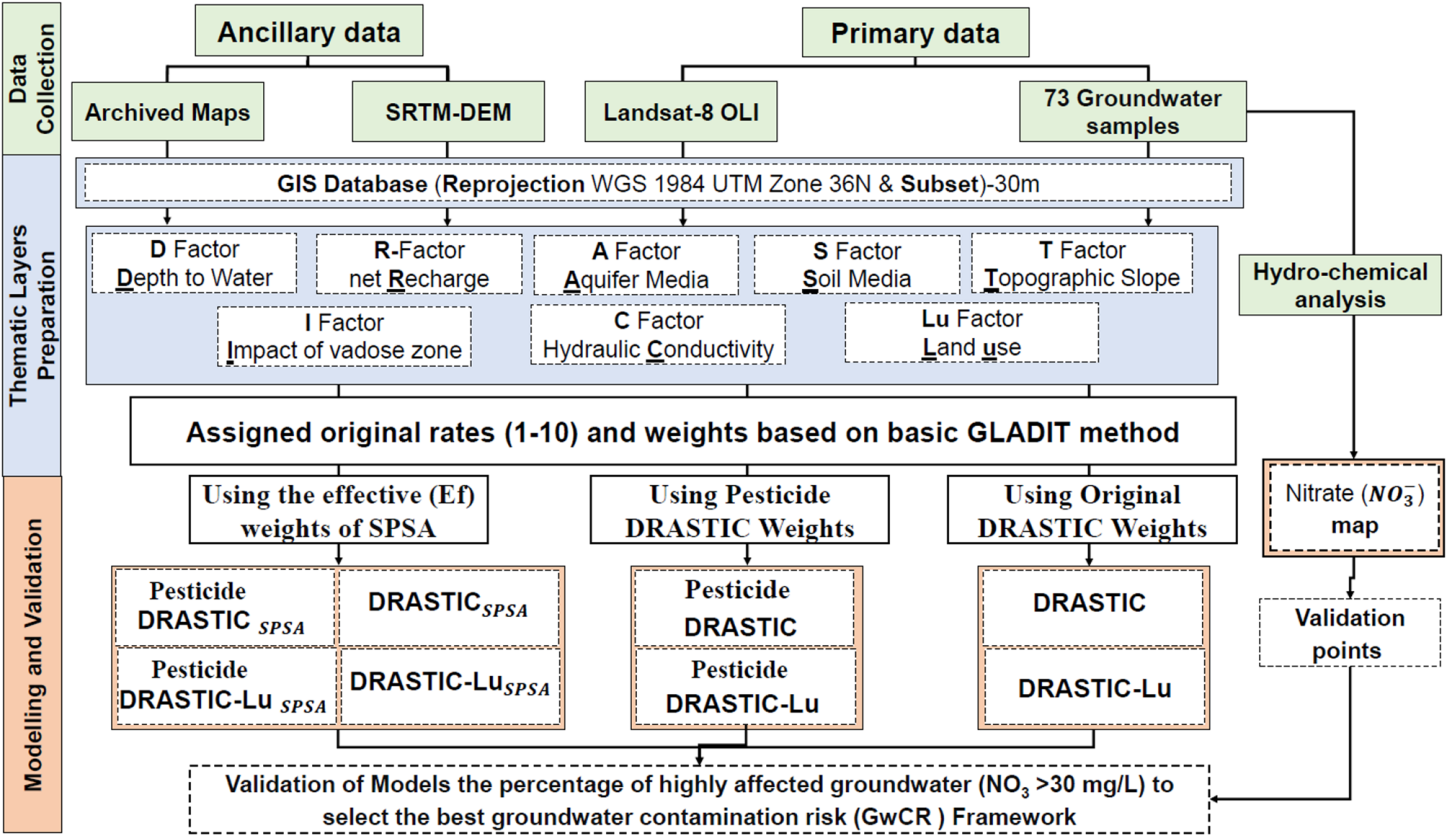
Flow diagram of the methodology implemented for groundwater vulnerability analysis.

Table 1 summarizes the datasets used in this research, while Fig. 4 outlines the four-step methodological framework: (1) developing a geodatabase, (2) generating thematic layers from satellite imagery, fieldwork, and archival sources, (3) implementing standard GwCR-based approaches, such as DRASTIC, Pesticide DRASTIC, DRASTIC-Lu, and Pesticide DRASTIC-Lu, (4) refining GwCR maps by incorporating Single Parameter Sensitivity Analysis (SPSA) weights, and (5) validating the models to evaluate their effectiveness in pinpointing groundwater vulnerability in areas experiencing intense urbanization and/or agricultural activities.

**Table 1:** The specification of datasets acquired and utilized in this study.

Parameter	Datasets specification	Source
Depth to water	Field Observations for groundwater levels from water wells in 2020.	^45^
Net Recharge	Interpolate data from a published map	^50^
Aquifer Media	Interpolate data from a published map	^59^
Soil Media	Interpolate data from a published map	^60^
Topography (T)	One scene of digital elevation model (DEM) derived from SRTM global elevation data (1 Arc Second resolution, SRTM plus v3). Spatial Resolution (30 m).	https://earthexplorer.usgs.gov/
Impact of vadose zone/Unsaturated conditions	Interpolate data from a published map	^60,61^
Hydraulic Conductivity	Interpolate data as points from a published map.	^62^
Land Use (U)	Landsat-8: one scene (Path/Row: 176/39) of bands (visible blue, green, and red), near-infrared (NIR), short infrared (SWIR1), and short infrared (SWIR2)). Spatial Resolution (30 m).	https://earthexplorer.usgs.gov/
Nitrate (NO3) concentrations	Measured NO_3_ data from groundwater wells (**2020**)	Groundwater sampling during field survey by authors utilizing the GPS (2019) and^45^

**Fig. 5:**
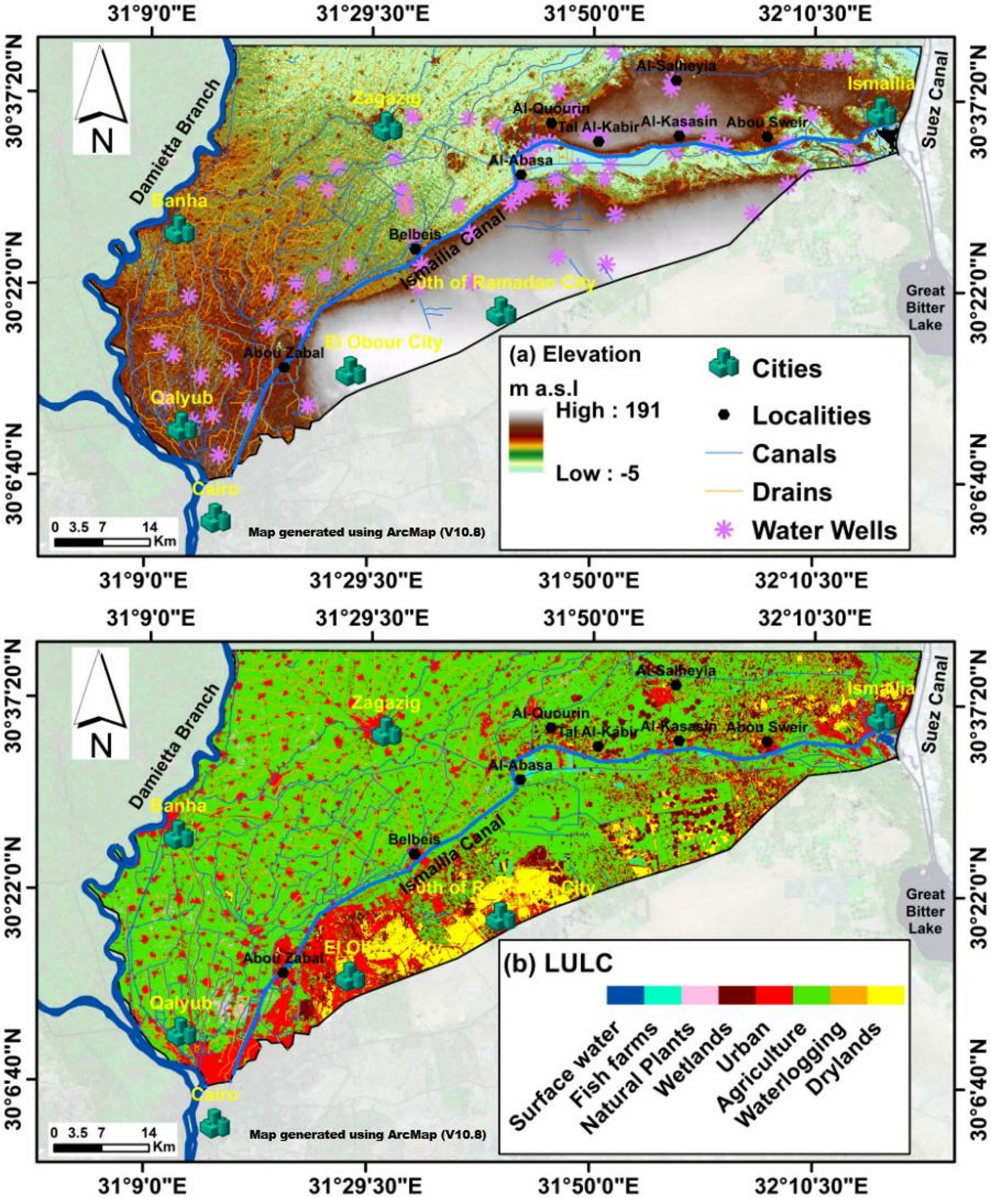
(**A**) SRTM-Digital elevation map (DEM) and (**B**) Land Use/Land Cover (LULC) of the study area.

**Fig. 6:**
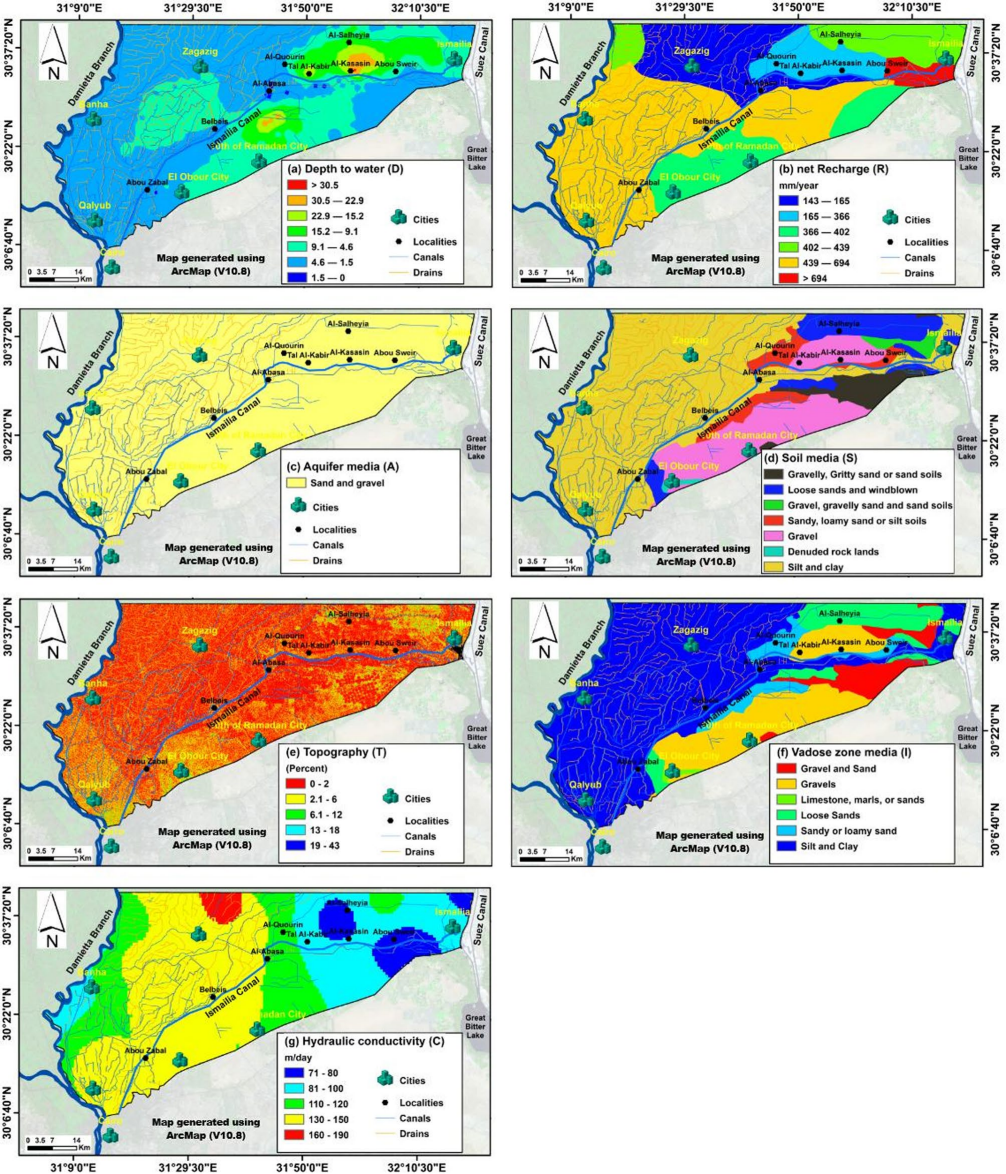
Distribution of the DRASTIC vulnerability conditioning factors in the area: (**A**) Depth to water table (**D**), (**B**) the annual net recharge net Recharge (**R**), (**C**) Aquifer media (**A**), (**D**) Soil media (**S**), (**E**) Topography (**T**), (**F**) Impact of vadose zone (**I**), (**G**) hydraulic Conductivity (**C**).

### Fieldwork and data Preparation

The primary data for this study comprised satellite imagery and field observations, supplemented by an extensive review of region-specific literature (Figs. 5 and 6; Table 1). A single cloud-free Landsat 8 Operational Land Imager (OLI) scene from 2020 was utilized, alongside a Shuttle Radar Topography Mission Digital Elevation Model (SRTM-DEM) scene covering the study area (Fig. 5A). Both datasets were freely obtained from the U.S. Geological Survey (USGS) EarthExplorer platform (https://earthexplorer.usgs.gov/, accessed on 15 May 2024), with their key specifications detailed in Table 1. For image preprocessing, only non-thermal bands (2, 3, 4, 5, 6, and 7) of the Landsat 8 scene were selected and spatially subset to delineate the study area (Fig. 1). These bands were subsequently calibrated to surface reflectance using radiometric correction and atmospheric compensation via the Fast Line-of-sight Atmospheric Analysis of Spectral Hypercubes (FLAASH) module within the ENVI 5.1 software environment.

**Fig. 7:**
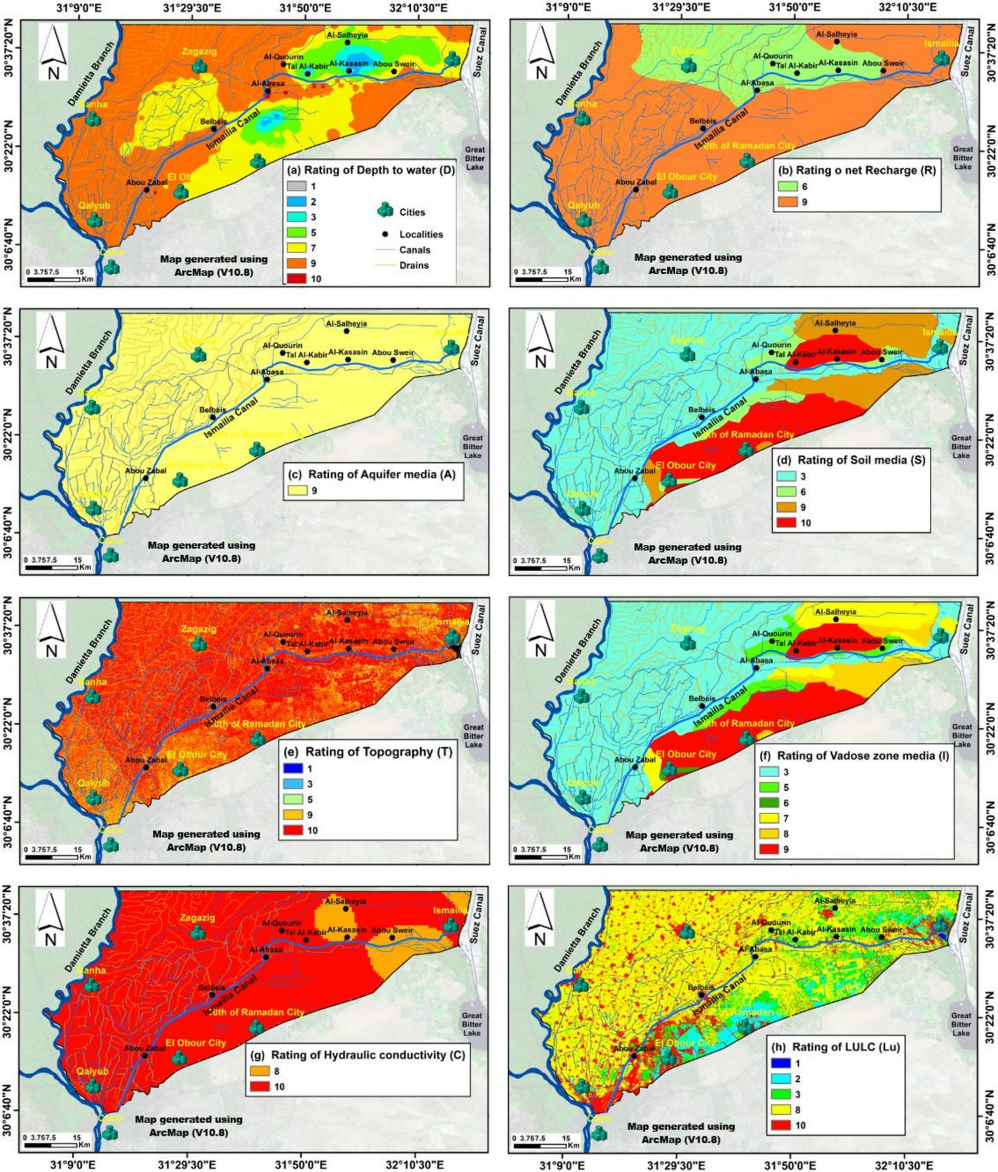
The thematic DRASTIC and DRASTIC-LU rated maps: (**A**) Depth to water Table, (**B**) Net Recharge, (**C**) Aquifer Media, (**D**) Soil Media, (**E**) Topography, (**F**) Impact of Vadose Zone, (**G**) Hydraulic Conductivity, and (**H**) LULC. For a 1 to 10 score rating of maps see Table 3.

During 2019, a total of 73 groundwater samples were collected from wells across the study area. Sampling followed the guidelines outlined by^56^ for water analysis. Groundwater samples were filtered and stored in pre-cleaned, high-density polyethylene one-liter containers with polyethylene closures. The containers were labeled, placed in a cooler, transported to the laboratory, and kept at or below 4 °C until further analysis. Electrical conductivity (EC), total dissolved solids (TDS), and pH were measured in situ using a portable Hach 44,600 Conductivity/TDS Meter (3.5-digit, accuracy ± 2%, range up to 20 mS/cm and 20 g/l) and a portable Hach P314 pH meter^57^. Nitrate (NO^3−^) concentrations were analyzed using the DX-500 Ion Chromatography system (USA), with an accuracy and precision of 5%. Sampling locations were precisely recorded with GPS equipment, as shown in Fig. 1B. Thematic imagery was converted to 30-meter raster layers in the UTM-WGS 1984, zone 36 N projection using the ArcMap (10.8) package established by ESRI^58^.

The DRASTIC vulnerability maps were developed by integrating diverse datasets. Seven parameters, listed in Table 1, were generated using ArcMap 10.8. The D—depth to water factor was created based on a water level map from^45^ using the Inverse Distance Weighted (IDW) interpolation tool (Fig. 6A). The R—net recharge map was constructed using annual precipitation, drainage surplus, and evapotranspiration data for the Nile Delta (Fig. 6B), following^50^. The A—aquifer media map utilized data from^59^. The S—soil map was derived from soil data provided by^60^. The T—topographic map was generated using slope percentages from Shuttle Radar Topography Mission-Digital Elevation Model (SRTM-DEM) data (Figs. 5 and 6E). The I factor (Fig. 6F) was determined by comparing^61^ with^60^. The C factor map was created by digitizing points from^44,62^ and applying IDW interpolation (Table 2). The Lu—land use map combined Landsat 8 (L8) with field surveys and high-resolution Google Earth imagery, using 70% of the data for training a maximum likelihood classifier and 30% for testing (Fig. 5B).

**Table 2:** Reported hydraulic conductivity values of the quaternary aquifer in the nile Delta (after^26,44^).

Reference	Hydraulic Conductivity (m/day)	References	Hydraulic Conductivity (m/day)
Shata & El Fayoumy (1970)	86	Leaven (1991)^88^	150
Farid (1980)	112	RIGW (1992)^89^	35–100
Mabrook et al., (1983)	72–108	Bahr (1995)^90^	25–40
Shahin (1983)	50	Sallouma & Gomaa (1997)^91^	23–65
Zaghloul (1985)	119	Sherif et al., (2012)^92^	36–240

### Standard DRASTIC models implementation

 The DRASTIC parameters were assigned ratings on a scale of 1 to 10, based on the available literature (Table 3), where a rating of 1 represents the least important range or media type, and a rating of 10 corresponds to the most important (Fig. 7). The ranges, ratings, and usage instructions are consistent for both the DRASTIC and Pesticide DRASTIC indices^24,36^. Similarly, the land use parameter, which has a weight of 5, is categorized into types, with each type assigned a specific rating value as detailed in Table 3^36,47^. Each DRASTIC parameter is given a weight (w) ranging from 1 to 5, indicating its relative significance, where a weight of 1 represents the least significant parameter and a weight of 5 corresponds to the most significant (Table 3). For the Pesticide DRASTIC and Pesticide DRASTIC-Lu, a variant of the DRASTIC system, a second weight is assigned to account for the impact of pesticide use (Table 3), with groundwater pollution potential primarily influenced by pesticides^24^. The DRASTIC and Pesticide DRASTIC indices were calculated using Eq. (1) while the DRASTIC-Lu and Pesticide DRASTIC-Lu were calculated using Eq. (2).

1 $$\rm{DRASTIC=D_rD_w+R_rR_w+A_rA_w+S_rS_w+T_rT_w+I_rI_w+C_rC_w}$$

2 $$\rm{DRASTIC-Lu=D_rD_w+R_rR_w+A_rA_w+S_rS_w+T_rT_w+I_rI_w+C_rC_w+LU_rLU_w}$$

Where D, R, A, S, T, I and C are the condition vulnerability parameters and Lu is the land use parameter. The subscript ‘r’ is the rating allocated to each range or media type and the subscript ‘w’ is the weight allocated to each parameter^24,47,63^.

**Table 3:** Description of the DRASTIC and DRASTIC-Lu factors and their ranges, ratings, and weighting values used in this study^7,24,46,47^.

Factor	Range	Rating	DRASTIC	Pesticide DRASTIC
**Depth to Water** **(** ***D*** **)** **(m)**	0.0-1.5	10	5	5
	1.5–4.6	9		
	4.6–9.1	7		
	9.1–15.2	5		
	15.2–22.9	3		
	22.9–30.5	2		
	> 30.5	1		
**Net Recharge** **(** ***R*** **)** **(mm/year)**	0.0-50.8	1	4	4
	50.8-101.6	3		
	101.6-177.8	6		
	177.8–254	8		
	> 254	9		
**Aquifer Media** **(A)**	Massive Shale	3	3	3
	Metamorphic/Igneous	4		
	Weathered Metamorphic/Igneous	5		
	Glacial Till	6		
	Bedded Sandstone, Limestone and Shale sequences	6		
	Massive Sandstone	6		
	Massive Limestone	8		
	Sand and Gravel	9		
	Basalt	10		
	Karst Limestone	10		
**Soil Media** **(** ***S*** **)**	Thin or absent	3	2	5
	Gravel	4		
	Sand	5		
	Peat	6		
	Shrinking and/or aggregated clay	6		
	Sandy loam	6		
	Loam	8		
	Silty loam	9		
	Clay loam	10		
	Muck	2		
	Non-shrinking and/or non-aggregated Clay	3		
**Topography** **(** ***T*** **)** **(% Slope)**	0–2	2	1	3
	2–6	3		
	6–12	4		
	12–18	5		
	> 18	6		
**Impact of the Vadose Zone Media** **(** ***I*** **)**	Confining layer	4	5	4
	Silt/Clay	5		
	Shale	6		
	Limestone	6		
	Sandstone	6		
	Bedded limestone. Sandstone. Shale	8		
	Sand and gravel with silt and clay	9		
	Karts limestone	2		
**Hydraulic Conductivity** **(** ***C*** **)** **(m/d)**	0.04–4.1	6	3	2
	4.1–12.3	6		
	12.3–28.5	8		
	28.5–40.7	9		
	40.7–81.5	10		
	> 81.5	2		
**Land Use** **(Lu)**	Urban areas	10	5	5
	Rural and industrial	9		
	Agricultural	8		
	Fallow/wetlands	3		
	drylands	2		
	Surface water/waterlogged	1		

### Accuracy assessment of DRASTIC models

#### Map removal sensitivity analysis (MRSA)

Map Removal Sensitivity Analysis (MRSA) was employed to evaluate the uncertainty level of the model outputs and determine the necessity of incorporating all DRASTIC parameters^36,64^. This method involves excluding one or more maps from the vulnerability assessment and calculating a variation index (Tables 4 and 5) using Eq. (3), as defined by^65^.

3 $$\:S=\left[\frac{\left(\frac{V}{N}\right)-\left(\frac{{V}^{{\prime\:}}}{n}\right)}{V}\right]*100$$

Where, S is the sensitivity associated with the removal of one map; V is the unperturbed vulnerability index (actual index obtained using all seven maps); V‵ is the perturbed vulnerability index (vulnerability index calculated using a lower number of maps); N is the number of maps used in the unperturbed vulnerability and n is the number of maps used in the perturbed vulnerability.

**Table 4:** Statistics of the One-map removal sensitivity analysis.

Model	Removed Map	Variation Index Percentage (VI %)			
		Mean	Min.	Max.	SD
**DRASTIC**	D	2.16	0.03	3.66	1.10
	R	1.21	0.27	1.90	0.66
	A	0.41	0.03	1.04	0.26
	S	1.13	0.27	1.66	0.50
	T	1.38	1.10	1.73	0.15
	I	0.89	0.19	2.37	0.56
	C	0.95	0.21	1.89	0.49
**Pesticide DRASTIC**	D	1.73	0.18	3.17	0.97
	R	1.36	0.60	1.94	0.56
	A	0.37	0.00	0.58	0.13
	S	1.00	0.35	2.08	0.44
	T	0.45	0.00	0.90	0.27
	I	0.65	0.03	1.07	0.38
	C	0.42	0.03	0.88	0.32
**DRASTIC-Lu**	D	1.36	0.01	2.71	0.65
	R	0.95	0.09	1.46	0.50
	A	0.19	0.00	0.61	0.11
	S	0.90	0.09	1.30	0.39
	T	1.10	0.89	1.38	0.08
	I	0.70	0.02	2.04	0.39
	C	0.51	0.02	1.21	0.26
	LU	1.24	0.67	2.04	0.35
**Pesticide DRASTIC-Lu**	D	1.11	0.01	2.31	0.58
	R	1.04	0.34	1.49	0.43
	A	0.19	0.00	0.50	0.16
	S	0.79	0.14	1.84	0.32
	T	0.23	0.01	0.67	0.12
	I	0.55	0.00	0.89	0.32
	C	0.40	0.03	0.72	0.18
	LU	1.03	0.40	1.79	0.35

**Table 5:** Multi-map removal sensitivity analysis.

Model	Removed Maps	Used Maps	Variation Index Percentage (VI %)			
			Mean	Min.	Max.	SD
**DRASTIC**	AI	DRSTC	0.86	0.06	3.02	0.79
	AIC	DRST	2.50	1.25	6.17	0.78
	ASIC	DRT	1.34	0.13	6.88	1.45
	RASIC	DT	3.93	0.19	8.30	2.41
	RASTIC	D	21.95	12.93	0.17	6.63
**Pesticide DRASTIC**	AC	DRSTI	0.83	0.08	1.75	0.57
	ATC	DRSI	1.65	0.15	2.72	0.61
	ATIC	DRS	1.34	0.03	3.63	1.14
	ASTIC	DR	1.96	0.05	8.13	1.34
	RASTIC	D	10.36	1.06	19.05	5.80
**DRASTIC-Lu**	AC	DRSTI-LU	0.72	0.00	2.13	0.48
	AIC	DRST-LU	1.07	0.25	4.29	0.83
	ASIC	DRT-LU	1.39	0.00	5.21	0.85
	RASIC	DT-LU	3.51	0.06	6.75	2.08
	RASTIC	D-LU	13.36	7.91	0.13	4.19
	RASTICL	D	18.97	9.51	0.06	4.52
**Pesticide DRASTIC-Lu**	AT	DRSIC-LU	0.49	0.02	1.25	0.23
	ATC	DRSI-LU	0.83	0.05	2.18	0.71
	ATIC	DRS-LU	1.47	0.06	2.76	0.62
	ASTIC	DR-LU	2.30	0.03	5.73	1.23
	ASTICL	DR	1.30	0.10	7.72	1.28
	RASTICL	D	7.79	0.06	16.16	4.08

#### Single parameter sensitivity analysis (SPSA)

The single parameter sensibility test was performed to check the influence of the DRASTIC parameters on the vulnerability index and compare the theoretical weights of such parameters with their effective weights. The effective weight of a parameter is a function of the other parameters as well as the weight assigned to such parameter by the DRASTIC system. The single parameter sensitivity analysis was carried out using Eq. (4)^66^.

A Single Parameter Sensitivity Analysis (SPSA) was applied to evaluate the influence of each parameter on the vulnerability model^66,67^ by comparing the theoretical weights with the Effective (Ef) weights assigned to each parameter (Table 6). The Ef weights were calculated using (4):

4 $$\:{\text{E}\text{f}}_{\text{w}}=\left(\frac{{P}_{ri}*{P}_{wi}}{V}\right)*100$$

Where, $\:{\text{E}\text{f}}_{\text{w}\:}$is the effective weight (%); P_ri_ is the rating of the respective parameter; P_wi_ is the weight of respective parameter; and V is the final vulnerability index^25,36,38^.

**Table 6:** Statistics analysis of single-parameter sensitivity analysis (SPSA).

Index	Parameter	Theoretical Weight	Theoretical Weight%	Effective Weight%				Effective (Ef) Weight
				Mean	Min	Max	SD	
**DRASTIC**	D	5	21.74	26.58	4.27	36.23	7.76	6.11
	R	4	17.39	7.05	2.90	12.66	3.97	1.62
	A	3	13.04	16.01	12.44	20.51	2.37	3.68
	S	2	8.70	7.52	4.35	12.66	2.98	1.73
	T	1	4.35	5.98	3.88	7.69	0.90	1.38
	I	5	21.74	16.85	10.87	28.48	5.78	3.87
	C	3	13.04	20.01	15.54	25.64	2.97	4.60
**Pesticide DRASTIC**	D	5	19.23	23.64	3.62	33.33	7.32	6.15
	R	4	15.38	6.15	2.63	10.70	3.35	1.60
	A	3	11.54	14.19	10.81	17.78	2.38	3.69
	S	5	19.23	16.46	9.87	26.74	6.17	4.28
	T	3	11.54	15.91	11.11	19.71	2.69	4.14
	I	4	15.38	11.82	7.89	19.25	3.79	3.07
	C	2	7.69	11.83	9.01	14.81	1.98	3.07
**DRASTIC-Lu**	D	5	17.86	21.33	3.18	31.47	5.74	5.97
	R	4	14.29	5.86	2.25	11.90	3.51	1.64
	A	3	10.71	12.88	10.08	16.78	1.48	3.61
	S	2	7.14	6.20	3.37	11.90	2.74	1.74
	T	1	3.57	4.82	2.87	6.29	0.57	1.35
	I	5	17.86	13.87	8.43	26.79	5.45	3.88
	C	3	10.71	16.10	12.61	20.98	1.85	4.51
	LU	5	17.86	18.93	4.93	26.79	6.35	5.30
**Pesticide DRASTIC-Lu**	D	5	16.13	19.36	2.81	28.66	5.50	6.00
	R	4	12.90	5.22	2.08	10.15	3.03	1.62
	A	3	9.68	11.65	8.99	15.29	1.53	3.61
	S	5	16.13	13.86	7.81	25.38	5.81	4.30
	T	3	9.68	13.07	8.33	17.20	1.75	4.05
	I	4	12.90	9.93	6.25	18.27	3.67	3.08
	C	2	6.45	9.71	7.49	12.74	1.27	3.01
	LU	5	16.13	17.20	4.31	25.00	5.96	5.33

The modified DRASTIC_SPSA_ and Pesticide DRASTIC_SPSA_ indices were calculated using Eq. (5) while the DRASTIC-Lu_SPSA_ and Pesticide DRASTIC-Lu_SPSA_ were calculated using Eq. (6).

5 $$\rm{DRASTIC_{SPSA}=D_rD_{Ef}+R_rR_{Ef}+A_rA_{Ef}+S_rS_{Ef}+T_rT_{Ef}+I_rI_{Ef}+C_rC_{Ef}}$$

6 $$\rm{DRASTIC-Lu_{SPSA}=D_rD_{Ef}+R_rR_{Ef}+A_rA_{Ef}+S_rS_{Ef}+T_rT_{Ef}+I_rI_{Ef}+C_rC_{Ef}+LU_rLU_{Ef}}$$

Where, the subscript ‘r’ is the rating allocated to each range or media type and the subscript ‘Ef’ is the effective weight allocated to each parameter^24,47^. See Table 7.

**Table 7:** The statistical summary of the vulnerability indexes’ results.

Index	Min.	Max.	Mean	Std. Dev.
**DRASTIC**	124	210	170.68	17.09
**DRASTIC-Lu**	139	260	207.44	16.64
**Pesticide DRASTIC**	135	241	191.38	23.00
**Pesticide DRASTIC-Lu**	149	291	228.13	21.15
**DRASTIC**_**SPSA**_	131.71	210.94	177.62	12.88
**DRASTIC-Lu**_**SPSA**_	145.97	261.29	214.01	14.69
**Pesticide DRASTIC**_**SPSA**_	143.32	241.8	199.20	17.46
**Pesticide DRASTIC-Lu**_**SPSA**_	152.41	292.07	235.43	17.28

#### Models validation

The accuracy of susceptibility models was assessed using the simple frequency ratio and relative frequency distribution (RFD) methods. A critical step involved evaluating the agreement between polluted groundwater (PGw) samples, with high nitrate concentrations (> 30 mg/L), and areas classified as highly vulnerable^68^. Accordingly, all generated maps were categorized into five vulnerability levels—very low, low, moderate, high, and very high—using the geometrical classification approach in ArcGIS software^69^. The RFD method offered another measure of the consistency of the vulnerability maps beyond traditional metrics (Table 8). The consistency of the prediction map was confirmed by calculating the RFD of affected infrastructure occurrences across various GwCR zones^70^.

**Table 8:** Variation of polluted groundwater (PGw) relative frequency distribution (RFD) for DRASTIC models validation with hazard vulnerability categories distribution.

Vulnerability Class	Attributes	DRASTIC	DRASTIC-Lu	Pesticide DRASTIC	Pesticide DRASTIC-Lu	DRASTIC_SPSA_	DRASTIC-Lu_SPSA_	Pesticide DRASTIC_SPSA_	Pesticide DRASTIC-Lu_SPSA_
Very low	Area (km2)	21.94	7.28	29.46	4.40	15.16	6.76	13.09	3.76
	PGw (%)	7	21	11	12	12	15	11	8
	RFD	0.05	0.49	0.05	0.42	0.17	0.37	0.12	0.34
Low	Area (km2)	23.70	24.82	37.88	26.70	8.57	6.91	31.10	6.62
	PGw (%)	13.17	5.8	45.77	8.62	10.34	9.87	6.74	13.01
	RFD	0.08	0.04	0.18	0.05	0.26	0.24	0.03	0.30
Moderate	Area (km2)	19.89	2.49	7.97	27.14	13.23	8.44	19.03	11.59
	PGw (%)	30.25	1.1	24.76	24.45	2.35	5.49	25.24	4.39
	RFD	0.23	0.08	0.46	0.13	0.04	0.11	0.19	0.06
High	Area (km2)	7.17	30.04	7.63	16.19	34.41	32.86	6.26	44.80
	PGw (%)	23.67	7.17	13.79	25.55	35.58	16.77	21.16	26.68
	RFD	0.50	0.18	0.27	0.23	0.22	0.09	0.49	0.10
Very high	Area (km2)	27.30	35.37	17.06	25.56	28.64	45.04	30.52	33.24
	PGw (%)	26.18	42.32	5.02	29	39.66	52.98	36.21	45.61
	RFD	0.14	0.21	0.04	0.17	0.30	0.20	0.17	0.21
Consistency of vulnerability levels		0.87	0.46	0.77	0.54	0.56	0.39	0.85	0.36

## Results

### Geospatial analysis of DRASTIC parameters and land use

The D factor is a critical determinant for assessing groundwater pollution risk^24^. Depth values in the study area range from 0 to over 30.5 m (Fig. 6A), with the most common depth interval, 1.5–4.6 m, assigned a rating of 9. The shallowest depth range (0.0–1.5 m), rated at 10, is found primarily along the Ismailia Canal between Al-Abasa and Abu Suwayr. In contrast, the deepest range (22.9–30.5 m), rated at 2, is located north of the Ismailia Canal, between Al-Kasasin, Abu Suwayr, and Al-Salheyia, and extends southward near Al-Abasa (Fig. 7A). The D factor was thus classified into seven categories, with ratings from 1 to 10, as shown in Fig. 7A.

The N factor measures surface water volume per unit area from rainfall and artificial sources^71^. Recharge rates range from 143 to over 694 mm/year (Fig. 6B) and are classified into two groups (Fig. 7B) based on the DRASTIC rating system^24^. The aquifer media parameter characterizes the properties of the aquifer materials influencing pollutant attenuation^72^. The Mit Ghamr Formation, predominantly composed of sand and gravel (Fig. 6C), features high porosity, excellent drainage, and transmission capacity^45^, earning a rating of 8 (Fig. 7C).

Soil acts as the top layer where weathering occurs, regulating water infiltration and pollutant transport into the vadose zone^72^. Coarse-grained soils like gravel, gravelly sand, and loose sand (Fig. 6D) provide efficient drainage^45^, leading to high ratings for pollutant transfer. In contrast, silt and clay soils restrict water flow, reducing contamination risk and receiving lower ratings. The S factor was categorized into four classes with ratings from 3 to 10 (Fig. 7D).

The slope factor (T) influences surface pollutant infiltration into groundwater. Low slopes in the southern and northwestern areas enhance infiltration, increasing groundwater contamination risk (Fig. 6E). The region features low relief with a gentle northward slope and a rolling terrain that rises to a moderately elevated southern plateau^73,74^. Gentle slopes allow for higher infiltration, while steep slopes decrease water retention and infiltration rates (Nahin et al., 2019). The slope values were rated according to^24^, as shown in Table 3; Fig. 7E.

The I factor (Fig. 6F) represents the unsaturated zone between the surface and the aquifer^24^. Hydrogeological units were scored between 3 and 9 (Fig. 7F). The C factor determines groundwater flow rate (Fig. 6G), affecting contaminant dispersion in the aquifer^72^. High hydraulic conductivity (> 80 m/day) aquifers are rated at 10, while lower-conductivity media receive an 8 (Fig. 7G).

The U factor assesses land cover types and their impact on groundwater quality, particularly in urban settings^36,46,75^. Eight land cover categories were identified: agriculture, fish farms, urban areas (including roads and cities), drylands, wetlands, surface water bodies, and waterlogged areas (Fig. 5B). Agricultural land dominates, followed by urban areas. The U factor map, using^10^ rating system, assigns vulnerability scores to land cover types (Table 3). Urban areas have the highest vulnerability score of 10 (Fig. 7H), followed by agriculture (8), wetlands (3), drylands (2), and surface and waterlogged bodies (1).

### Standard DRASTIC vulnerability mapping

**Fig. 8:**
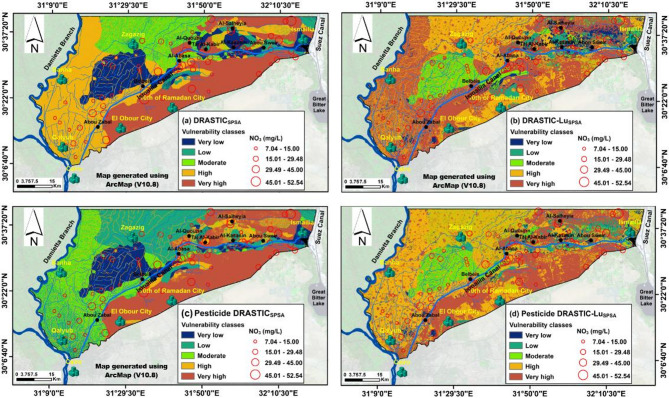
Typical groundwater contamination risk (GwCR) maps of the studied aquifer developed using: (**A**) DRASTIC, (**B**) DRASTIC-Lu, (**C**) Pesticide DRASTIC, and (**D**) Pesticide DRASTIC-Lu, overlaid with measured NO3 concentrations.

The seven DRASTIC parameters, along with the land use parameter, were integrated within a GIS environment to calculate the standard DRASTIC, Pesticide DRASTIC, DRASTIC-Lu, and Pesticide DRASTIC-Lu indices. These indices were used to evaluate the potential for groundwater contamination near the Ismailia Canal in the Eastern Nile Delta, considering the hydrogeological characteristics of the area, the effects of pesticide application, and the impact of various land use types. Figure 8 presents a groundwater vulnerability map classified according to DRASTIC index values, where higher values indicate greater groundwater vulnerability^24,36,46,66^. The five vulnerability classes, namely very low, low, moderate, high, and very high, were provided in square kilometers (Table 8). The DRASTIC index classified approximately 21.94 km², 23.7 km², 19.89 km², 7.17 km², and 27.3 km² of the study area into very low, low, moderate, high, and very high vulnerability zones to pollution risks, respectively (Fig. 8; Table 8). Highly vulnerable areas were mostly concentrated in the southern parts across all maps. However, these zones were significantly reduced in the Pesticide DRASTIC model, especially in the northern region between Al-Salheyia and Al-Kasasin settlements. The inclusion of the Lu factor increased vulnerability from moderate in the standard DRASTIC model too high in the DRASTIC-Lu model, particularly in the western region near the Damietta branch (Figs. 8a-b). Similarly, in the Pesticide DRASTIC model, vulnerability rose from low to moderate in the same western areas upon adding the Lu factor (Figs. 8C-D).

### Modified DRASTIC models mapping through sensitivity analysis

The one- MRSA reveals that the D factor is the most influential across all models, with the highest mean variation indices: 2.16% for DRASTIC, 1.73% for pesticide DRASTIC, 1.36% for DRASTIC-LU, and 1.11% for pesticide DRASTIC-LU, due to its high theoretical weight (5). In contrast, the A factor exhibits the lowest mean variation index, contributing no variability (CV% = 0). Although the D map removal results in the greatest variation in the DRASTIC index, other factors like topography, despite its low theoretical weight (1), also significantly affect the index. The R and S factors show mean variation indices of 1.38%, 1.21%, and 1.13%, respectively. The index shows moderate sensitivity to hydraulic conductivity (0.95%) and vadose zone impact (0.89%), even with their high weights (5). For pesticide DRASTIC, the index is highly sensitive to net recharge (1.36%) and soil media (1.0%), with moderate sensitivity to the vadose zone impact (0.65%), topography (0.45%), and hydraulic conductivity (0.42%). In DRASTIC-Lu, the Lu removal results in the highest sensitivity (1.24%), followed by topography (1.10%), while moderate sensitivity is seen for net recharge (0.95%), soil media (0.90%), vadose zone impact (0.70%), and hydraulic conductivity (0.51%). For pesticide DRASTIC-LU, variation indices are generally similar, differing by less than 1%, though net recharge (1.04%) and land use (1.03%) have the highest impact due to their high weights. Moderate sensitivity is noted for soil media (0.79%) and vadose zone impact (0.55%), while hydraulic conductivity (0.40%) and topography (0.23%) show lower sensitivity. Table 5 summarizes variation indices when multiple layers are removed simultaneously. For DRASTIC, the lowest variation occurs with the removal of A and I (mean VI% = 0.86). In pesticide DRASTIC and DRASTIC-Lu, removing A and C layers yields mean VI% values of 0.83 and 0.72, respectively. For pesticide DRASTIC-Lu, the minimum variation is associated with excluding aquifer media and topography (mean VI% = 0.49). Excluding more layers, especially D, R, and S for DRASTIC and pesticide DRASTIC, and D, Lu, and R for DRASTIC-Lu and pesticide DRASTIC-Lu, markedly increases the variation indices, impacting vulnerability assessments.

Statistical SPSA analyses were conducted to evaluate the Ef weight of each DRASTIC parameter, along with the land use, including the DRASTIC_SPSA_, Pesticide DRASTIC_SPSA_, DRASTIC-Lu_SPSA_, and Pesticide DRASTIC-Lu_SPSA_ indices. Table 4 presents the theoretical weights assigned by the DRASTIC system, the corresponding theoretical percentages, the mean effective weights, as well as the minimum and maximum values and standard deviations for the relevant vulnerability indices. As shown in Table 4, the calculated effective weights of the DRASTIC parameters differed from their theoretical values. Specifically, for all indices, the effective weights of D, A, T, and C exceeded the theoretical weights assigned by the DRASTIC system. Additionally, the effective weight of the Lu parameter also surpassed its theoretical value. In contrast, the effective weights of the R, S, and I parameters were lower than their theoretical counterparts for the indices examined. Among the parameters, D emerged as the most influential factor in the vulnerability assessment, with mean effective weights of 26.6%, 23.6%, 21.3%, and 19.4%, compared to theoretical weights of 21.7%, 19.2%, 17.9%, and 16.1% for DRASTIC, pesticide DRASTIC, DRASTIC-Lu, and pesticide DRASTIC-Lu, respectively. On the other hand, the R factor exhibited notably lower effective weights, with values of 7.05%, 6.15%, 5.86%, and 5.22% for the respective indices, as compared to theoretical weights of 17.4%, 15.4%, 14.3%, and 12.9%. Based on the mean effective weights (%), the significance of these parameters varied across the indices, as indicated by the following sequences: D > C > I > A > S > R > T for DRASTIC_SPSA_; D > S > T > A > I > C > R for pesticide DRASTIC_SPSA_; D > Lu > C > I > A > S > R > T for DRASTIC-Lu_SPSA_; and D > Lu > S > T > A > I > C > R for pesticide DRASTIC-Lu_SPSA_.

**Fig. 9:**
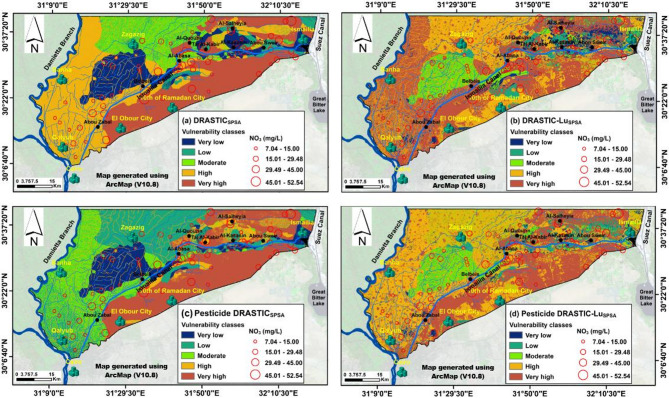
Modified GwCR maps of the studied aquifer developed using the effective (Ef) weights of single-parameter sensitivity analysis (SPSA) method: (**A**) DRASTIC_SPSA_, (**B**) DRASTIC-Lu_SPSA_, (**C**) Pesticide DRASTIC_SPSA_, and (**D**) Pesticide DRASTIC-Lu_SPSA_, overlaid with measured NO3 concentrations.

Applying effective weights led to an expansion of the high vulnerability zone in the DRASTIC_SPSA_ vulnerability map, reducing the extent of the moderate vulnerability zone in the standard DRASTIC model (Figs. 8A and 9A). Similarly, the very high vulnerability zone in the DRASTIC-Lu_SPSA_ map expanded at the expense of the high vulnerability zone in the DRASTIC-Lu model (Figs. 8B and 9B). Additionally, in the Pesticide DRASTIC_SPSA_ map, the moderate vulnerability zone increased, reducing the low vulnerability zone’s area in the Pesticide DRASTIC map (Figs. 8C and 9C). Meanwhile, in the Pesticide DRASTIC-Lu_SPSA_ map, the high vulnerability zone expanded, decreasing the extent of the moderate vulnerability zone in the Pesticide DRASTIC map (Figs. 8D and 9D). This trend can be attributed to the high-ranking values assigned to the urban and agricultural classes, accompanied by their high effective weights (%). This is likely due to the predominance of agricultural land in the study area, a finding that aligns with the results of Arafa et al.^34^.

### DRASTIC vulnerability validation

Overall, the results indicate that each model produces distinct outcomes regarding the ranges of groundwater vulnerability and their spatial distribution. For example, the standard DRASTIC vulnerability index yields a mean value of approximately 124, whereas the Pesticide DRASTIC-Lu_SPSA_ model shows the highest value (Table 7). The spatial distribution of highly vulnerable zones in the standard DRASTIC map generally correlates with that of the Pesticide DRASTIC map. However, differences are observed in the distribution of low and very low vulnerability areas within the DRASTIC-Lu map based on GwCR. Therefore, it is essential to assess the accuracy of these model predictions.

**Fig. 10:**
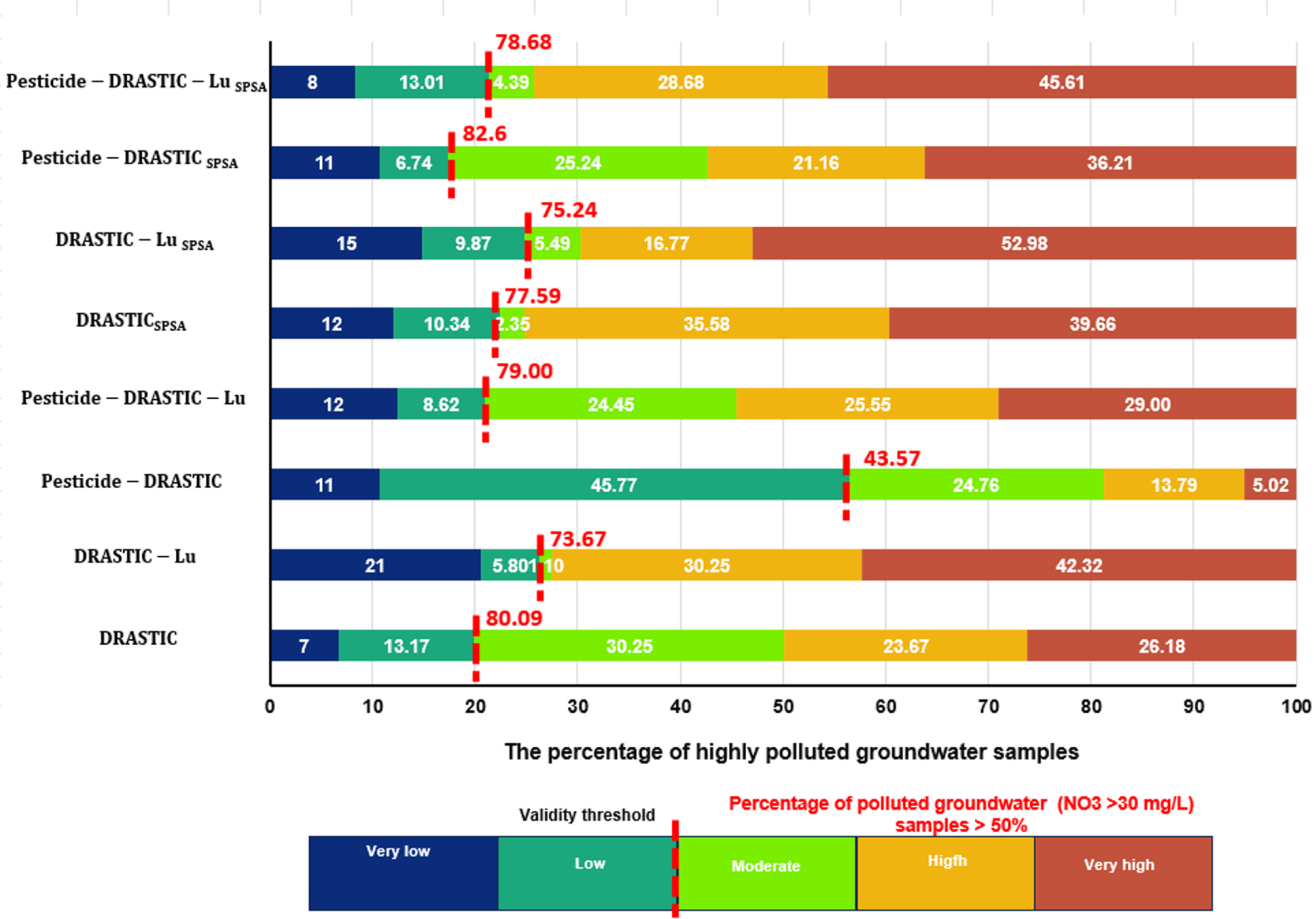
The clustered graph shows the percentage of highly affected groundwater (NO_3_ > 30 mg/L) samples in various classes of DRASTIC vulnerability models.

Nitrate concentrations in groundwater are predominantly influenced by human activities, as nitrates do not naturally occur in groundwater but are primarily linked to agricultural practices, such as the use of fertilizers and pesticides^76^. Nitrates are often used as a reliable indicator of contaminant migration from the surface to the groundwater^36,77,78^. As depicted in Fig. (9), the highest concentrations of NO_3_ were observed in the eastern part of the study area and south of the Ismailia Canal (Figs. 8 and 9), where nitrate levels exceeded the guideline value of 30 mg/L for irrigation water^79^. A GIS-based statistical overlay analysis was conducted to assess the consistency of vulnerability maps with actual polluted points. The validation hypothesis posits that the vulnerability map is reliable if more than 50% of the PGw points fall within at least moderate vulnerability zones (Fig. 10), with a preference for higher susceptibility regions (Youssef et al., 2021). The results showed that 82.6%, 80.09%, 79%, 78.68%, 77.59%, 75.24%, 73.67%, and 43.57% of the PGw samples were situated within the moderate to very high vulnerability zones across the Pesticide DRASTIC _SPSA_, DRASTIC, Pesticide DRASTIC-Lu, Pesticide DRASTIC-Lu_SPSA_, DRASTIC_SPSA_, DRASTIC-Lu- _SPSA_, DRASTIC-Lu, and Pesticide DRASTIC maps, respectively (Fig. 10). Of these models, the RFD values notably surpassed 0.8 for DRASTIC and Pesticide DRASTIC_SPSA_, measuring approximately 0.87 and 0.85, respectively, within the moderate to highly vulnerable zones (Table 8). These values indicate the reliability and consistency of the vulnerability assessments, aligning with findings from^80,81^. Consequently, this study demonstrates the improved accuracy of the Pesticide DRASTIC_SPSA_ vulnerability model in refining the GwCR map for the study area.

## Discussion

Groundwater contamination is a significant consequence of the water-food nexus in urbanized riverine areas^10,17^. The DRASTIC index has been used to evaluate groundwater’s intrinsic vulnerability to pollution from human activities, incorporating geological, hydrological, and hydrogeological factors. In the Eastern Nile Delta, intensive human and agricultural activities have substantially degraded groundwater quality, presenting serious environmental and soil risks. While extensive research has focused on water quality for drinking and sanitation^11^, studies using DRASTIC-based GwCR prediction models remain scarce, particularly in rapidly urbanizing riverine areas. Agriculture, characterized by extensive fertilizer and pesticide use and reliance on traditional irrigation, dominates these regions. To assess pesticide-related pollution risks, this study applied both the DRASTIC model and its pesticide-specific variant, enhancing them by integrating land use as an additional input parameter. When the impact of pesticide application was factored in using the Pesticide DRASTIC index, the values of the DRASTIC index increased, highlighting the potential for pesticide-induced groundwater contamination. Furthermore, the inclusion of the land use parameter in both the DRASTIC and Pesticide DRASTIC indices resulted in higher vulnerability values, emphasizing that groundwater susceptibility to pollution is strongly influenced by land use.

Generating a hazard vulnerability map without proper validation renders it scientifically meaningless^82^. Among all models, the Pesticide DRASTIC_SPSA_ model was the most effective in refining the GwCR map for the study area. This map revealed that approximately 36.21 km², 6.26 km², 19.03 km², 31.0 km², and 13.09 km² of the study area fall within very high, high, moderate, low, and very low susceptibility zones, respectively (Fig. 9C; Table 8). The moderate vulnerability zone, primarily located in the western part of the area, is characterized by a 5–20 m thick silt and clay layer^14^ that overlies the principal aquifer. In contrast, the high and very high vulnerability zones are concentrated to the north and south of the Ismailia Canal, where the protective clay layer is absent, unsustainable groundwater extraction in regions of moderate vulnerability could heighten pollutions risks. These zones are also distinguished by shallow groundwater depths and a vadose zone composed predominantly of sandy facies^8^. Notably, the area classified as very highly vulnerable increased from 27.3 km² in the original DRASTIC model to approximately 30.52 km² under the Pesticide DRASTIC-SPSA model (Table 8). These results suggest that soil media play a significant role in groundwater pollution potential. The silt and clay layers throughout most of the study area reduce soil permeability, restrict contaminant migration, and enhance attenuation processes^72,81^. Additionally, organic matter in the surface silt and clay layers, derived from decayed plant and animal material, aids in pesticide attenuation^83,84^.

The results are consistent with the outcomes of sensitivity analyses conducted to validate the vulnerability maps. The map removal sensitivity analysis confirmed that the DRASTIC parameters were sufficiently independent and collectively representative for evaluating groundwater pollution vulnerability, with no individual parameter identified as redundant. Among these parameters, SPSA analyses demonstrated that D factors exerted the greatest influence on vulnerability assessment, corroborating the findings of Metwally et al.^85^. Conversely, the pesticide-specific DRASTIC model exhibited heightened sensitivity to net recharge (1.36%) and soil media (1.0%), along with moderate sensitivity to the impact of the vadose zone (0.65%), topography (0.45%), and hydraulic conductivity (0.42%). This pattern may reflect the influence of the aquifer matrix and the overlying confining layer, as similarly observed in surface water canal systems within the El Fayoum Depression, Western Desert, Egypt, by Gad and El-Hattab^86^. Additionally, Ouedraogo et al.^87^ reported that removing the I, D, C, and R parameters in African aquifer systems significantly altered vulnerability mapping outcomes. These findings underscore the importance of including all pesticide DRASTIC parameters in vulnerability assessments to ensure accuracy, in alignment with the SPSA-derived effectiveness weights.

The preceding analysis underscores the synergistic impact of physiographical, hydrogeological, and anthropogenic factors on the aquifer’s potential vulnerability to human-induced contamination. Based on these insights, several key policy recommendations can be advanced to support sustainable groundwater management. First, it is imperative for policymakers to incorporate scientifically robust vulnerability maps—particularly those generated using the Pesticide DRASTIC-SPSA model—into spatial planning and land-use decision-making. These maps effectively classify areas according to varying degrees of susceptibility, ranging from very low to very high, thereby enabling the implementation of targeted mitigation strategies in high-risk zones. For example, in areas of elevated vulnerability adjacent to the Ismailia Canal, stringent regulation of agrochemical use and unsustainable groundwater abstraction is crucial to curbing further deterioration^10,13^. Consequently, zones identified as highly vulnerable should be prioritized for preventive measures, including the imposition of land-use restrictions and the deployment of protective strategies to safeguard groundwater quality^34^. These strategies may include the establishment of buffer zones, limitations on pesticide application, and the adoption of modern irrigation techniques designed to minimize contaminant transport via recharge. Furthermore, public awareness initiatives and community-based educational programs focused on sustainable agricultural practices and the implications of groundwater pollution are essential for achieving long-term resilience. This study also introduces a practical and adaptable modeling framework for assessing contamination potential in densely populated regions subjected to diverse anthropogenic pressures. The results not only contribute to informed water and food security planning but also emphasize the need for community engagement to mitigate salinization and associated health risks. This integrated approach offers a transferable methodology for groundwater vulnerability assessment in other regions with comparable hydrogeological and socio-economic settings.

## Conclusion

Groundwater contamination is a critical concern in rapidly urbanizing riverine areas, particularly where surface water is scarce or polluted. This study employed the DRASTIC-based GwCR framework to evaluate standard and land use–integrated DRASTIC and Pesticide DRASTIC models, along with their SPSA-optimized variants, to assess groundwater vulnerability around the Ismailia Canal. Findings highlight that agricultural activities—particularly fertilizer and pesticide application—are the primary sources of contamination. The Pesticide DRASTIC_SPSA_ model demonstrated superior performance, with 82.6% of PGw samples located in moderate to very high vulnerability zones and an RFD exceeding 0.8, indicating high reliability. This model delineated 36.21 km² as very high, 6.26 km² as high, 19.03 km² as moderate, 31.0 km² as low, and 13.09 km² as very low vulnerability. Highly vulnerable areas are concentrated north and south of the canal, where protective clay layers are absent, groundwater is shallow, and the vadose zone is sandy. Compared to the original DRASTIC model, the very high vulnerability zone expanded from 27.3 km² to 30.52 km². SPSA analysis revealed the following parameter weight hierarchy for the Pesticide DRASTIC model: D > S > T > A > I > C > R. One-MRSA analysis showed highest sensitivity to net recharge (1.36%) and soil media (1.0%), with moderate effects from vadose zone (0.65%), topography (0.45%), and conductivity (0.42%). Omitting key parameters like D, A, and S introduced significant variability. The proposed framework is adaptable to other urban riverine regions with comparable hydrogeological and socio-economic conditions. Future studies should incorporate machine learning–enhanced DRASTIC models, integrating variables such as pumping rates, land cover change, and integrated electrical conductivity (IEC) to improve both qualitative and quantitative groundwater risk assessments.

## Data Availability

The datasets used and/or analysed during the current study available from the corresponding author on reasonable request.
